# Serum interleukin 17A and interleukin 17F in children with inflammatory bowel disease

**DOI:** 10.1038/s41598-020-69567-x

**Published:** 2020-07-28

**Authors:** Paulina Krawiec, Elżbieta Pac-Kożuchowska

**Affiliations:** Department of Paediatrics and Gastroenterology, Medical University of Lublin, Al. Racławickie 1, 20-059 Lublin, Poland

**Keywords:** Chemokines, Inflammation, Biomarkers, Paediatric research, Gastrointestinal diseases

## Abstract

Interleukin 17A (IL-17A) and interleukin 17F (IL-17F) appear to play important role in pathogenesis of some autoimmune diseases. However, their role in inflammatory bowel disease (IBD) has not been yet fully elucidated. We aimed to determine serum IL-17A and IL-17F in children with IBD and to assess their association with IBD activity. Recruited children underwent blood tests including complete blood count, C-reactive protein, erythrocyte sedimentation rate, IL-17A and IL-17F and stool sampling for calprotectin. The study group comprised 68 children with IBD, including 43 with ulcerative colitis and 25 with Crohn’s disease. Control group included 20 healthy children. IL-17A was significantly increased in children with IBD (median: 10.95 pg/ml; range: 0.65–200.54 pg/ml) compared to controls (median: 4.09 pg/ml; range: 0.67–26.20 pg/ml) (p = 0.002). IL-17A was significantly increased in patients with active phase of ulcerative colitis (median: 14.58 pg/ml; range: 0.65–200.54 pg/ml) compared to those in ulcerative colitis remission (median: 8.13 pg/ml; range: 1.61–58.56 pg/ml) (p = 0.04). There were no significant differences in IL-17A among patients with active and inactive Crohn’s disease (p = 0.18). IL-17F did not differ significantly between children with IBD (median: 15.11 pg/ml; range: 0.09–189.84 pg/ml) and controls (median: 11.56 pg/ml; range: 0.19–32.49 pg/ml) (p = 0.33). Our study suggests that interleukin 17A may diverse active phase from remission only in ulcerative colitis but not in Crohn’s disease.

## Introduction

Inflammatory bowel disease (IBD) is a group of chronic disorders of gastrointestinal tract with multifactorial and complex pathogenesis which has not been fully elucidated^1^. There are three subtypes of IBD including Crohn’s disease, ulcerative colitis and IBD-unclassified^2^. Typically, ulcerative colitis is characterized by continuous mucosal inflammation localized to the colon, while inflammatory infiltration in Crohn’s disease involves mucosal and submucosal layers of any region of the gastrointestinal tract^2^. IBD-unclassified is a form of IBD colitis with features that make uncertain the differentiation between ulcerative colitis and Crohn’s disease at diagnosis^2^.

Although these diseases constitute distinct entities, it is hypothesised that IBD in general may occur in genetically susceptible individuals as a result of impaired immune response and alteration of gut microbiota triggered by environmental factors^1^. Disruption of both innate and adaptive immune responses are considered to play role in the pathogenesis of IBD^3–5^. Several components of innate immunity response has been found to be defective in IBD including epithelial barrier integrity, autophagy, unfolded protein response, expression of antimicrobial peptides, microbial sensing^3–5^. Moreover, imbalance in adaptive immune response has been also highlighted as a key factor in IBD development. Alteration the Th1/Th2 balance in favour of Th1 immune response has been associated with Crohn’s disease, while shift towards Th2 immune response with ulcerative colitis^3–5^. Beside Th1 and Th2 immune responses, a role of Th17 cells has been emerged in the pathogenesis of IBD^3,5,6^. Th17 cells produce IL-17A, IL-17F, Il-21 and IL-22, which are involved in the orchestration of the inflammatory response^7^.

Improving knowledge of immune-inflammatory mechanisms in IBD development, including dysregulation between pro- ant anti-inflammatory cytokines, may improve clinical approach to IBD by identification of certain cytokines as a potential diagnostic tests or therapeutic targets^6^.

The interleukin 17 (IL-17) family of cytokines consists of six ligands from IL-17A to IL-17F and five receptors from IL-17RA to IL-17RE^8,9^. To date IL-17A has attracted considerable interest to researchers and is the most extensively studied member of the IL-17 family.

IL-17A was originally named cytotoxic T-lymphocyte-associated antigen 8 (CTLA8) and previously was known as IL-17^10,11^. The IL-17A gene is located on the short arm of chromosome 6 at position 12 (6p12)^11^. IL-17A is a 35-kDa disulfide-linked homodimeric glycoprotein which consists of 155 amino acids^11,12^. IL-17 is secreted by CD4 Th17 cells, CD8 Tc17 cells, γδ T cells, natural killer T cells, group 3 innate lymphoid cells (ILC3s), and “natural” Th17 cells^8,9^.

IL-17F is encoded by the gene localized in adjacent to IL-17A gene on chromosome 6p12 and is usually co-expressed with IL-17A by type 17 cells^8,12^. Among the IL-17 family subsets, IL-17F demonstrates the highest degree of homology with IL-17A^12^. Both IL-17 A and IL-17F may exist in a form of homodimers or IL-17A/F heterodimer^8^.

IL-17A has been shown to play a protective role in the host defence against pathogens through eliciting acute immune response at epithelial and mucosal barriers^8,9^, to take part in tissue healing after injury^8^ or to maintain the epithelial tight-junction barrier during inflammation^8^. However, unrestrained and excessive activation of IL-17A is one of the potential mechanism underlying autoimmunity, chronic inflammatory conditions and neoplasms^8^. It has been shown that IL-17F is involved in maintaining host’s barrier function^8^ and may play role in the immunopathogenesis of some chronic disorders^8^.

Although recently there has been a substantial increase of interest of the role of IL-17 family in the development of autoimmune and inflammatory disorders, the knowledge about IL-17 cytokines contribution in the pathogenesis of inflammatory bowel disease (IBD) is limited and conflicting research results provoke discussion in that field. However, an improved understanding of the role of IL-17A and IL-17F in IBD may lead to important clinical implications. Interleukins may serve as a novel potential biomarker of the disease or may be used to design therapeutic approaches in IBD. To the best of our knowledge there are only single studies in small groups of children with IBD assessing IL-17A in paediatric IBD with conflicting results and it appears to exist no study assessing IL-17F in children with IBD ^13,14^. The aim of the study was to evaluate serum IL-17 A and IL-17F concentration in children with IBD compared to healthy controls.

## Results

The study group comprised 68 children with IBD including 43 (63.2%) children with ulcerative colitis and 25 (36.8%) with Crohn’s disease. There was a slight female preponderance with 35 (51.5%) girls and 33 (48.5%) boys. Patients’ age ranged from 6.5 to 18 years (median: 14.25 years old; mean: 13.6 ± 3.1 years old).

The disease duration ranged from 0 to 8 years with mean 1.19 ± 1.85 years. There were 25 (36.8%) treatment naïve patients. The other 43 (63.2%) patients were on IBD treatment.

The most of patients with ulcerative colitis had pancolitis (30; 69.8%), followed by extensive colitis (7; 16.3%), left-sided colitis (4; 9.3%) and proctitis (2; 4.6%).

The most common location of Crohn’s disease was ileocolonic with the involvement of the upper gastrointestinal tract (11; 44%) and ileocolonic without the involvement of the upper gastrointestinal tract (7; 28%). Five patients (20%) had colonic Crohn’s disease and the latter 2 (8%) had Crohn’s disease limited to the distal 1/3 ileum.

The IBD was active in 39 (57.4%) patients (23 with ulcerative colitis and 16 with Crohn’s disease). PCDAI ranged from 0 to 65 points with median 30 points and mean ± SD 28 ± 21.9 points. PUCAI ranged from 0 to 75 points with median 20 points and mean ± SD 24.6 ± 22.9 points.

In the control group there were 20 children with functional abdominal pain including 12 (60%) girls and 8 (40%) boys aged from 4.5 to 17.5 years old (median: 12.25 years; mean: 11.9 ± 3.47 years old).

The IL-17A concentration in children with IBD ranged from 0.65–200.54 pg/ml with median 10.95 pg/ml and mean ± SD: 18.56 ± 27.77 pg/ml. Concentration of IL-17A was significantly increased in IBD patients compared to control group (median: 4.09 pg/ml; range: 0.67–26.20 pg/ml; mean ± SD: 6.93 ± 7.33 pg/ml) (p = 0.002).

Subgroup analysis, demonstrated in Table 1, revealed that IL-17A concentration was significantly higher in ulcerative colitis patients compared to controls (p = 0.003). No significant differences were found between Crohn’s disease patients and controls and between children with Crohn’s disease and ulcerative colitis.

**Table 1:** Serum concentration of IL-17A in children with IBD and controls.

		Ulcerative colitis	Crohn’s disease	Control group	Kruskal–Wallis test
Il-17A (pg/ml)	Mean ± SD	21.05 ± 32.36	13.83 ± 15.26	6.93 ± 7.33	H = 11.55 p = 0.003†
	Median (range)	12.59 (0.65–200.54)	8.76 (0.93–61.42)	4.09 (0.67–26.20)	

^†^p < 0.05 when comparing ulcerative colitis and controls.

**Figure 1:**
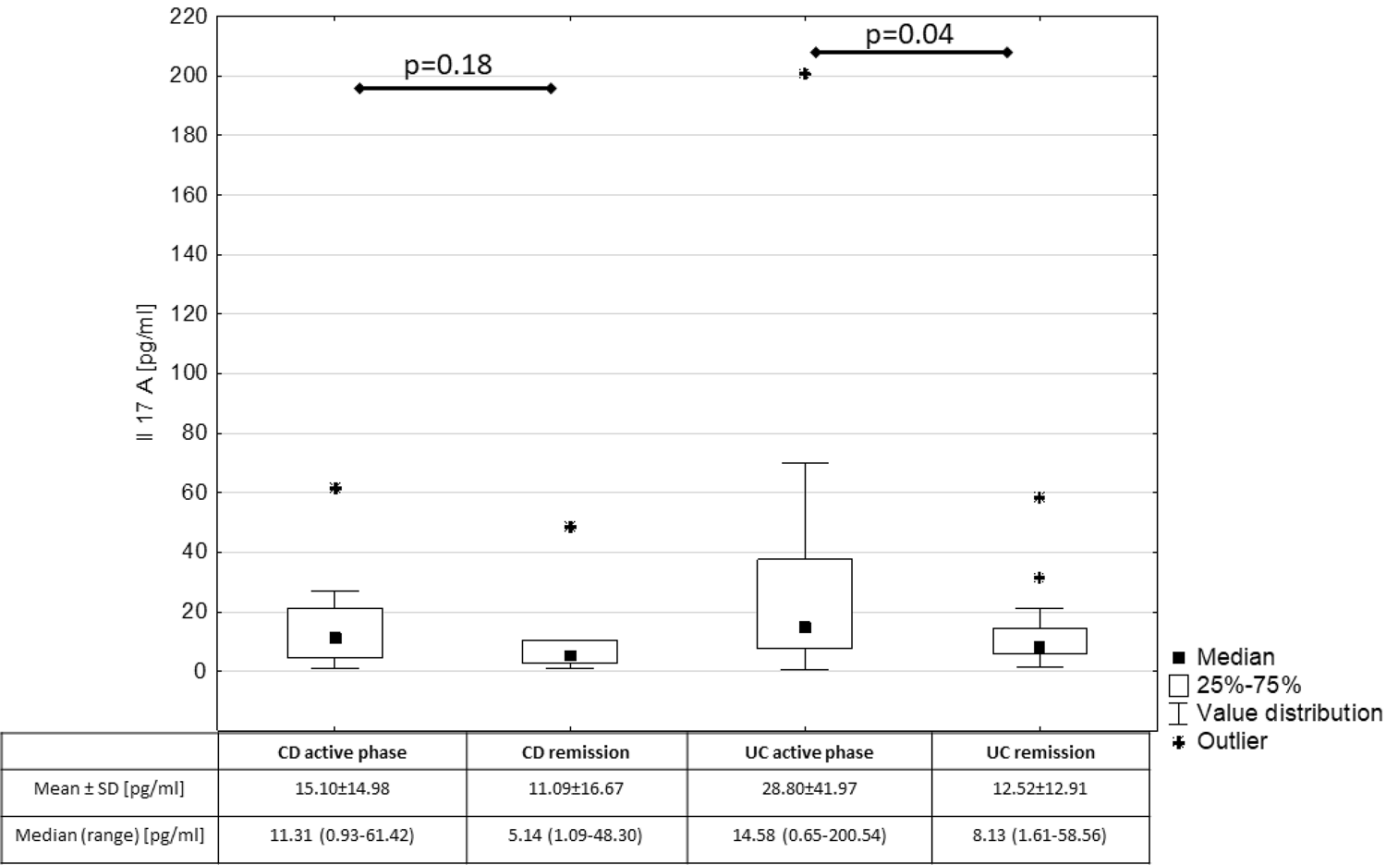
Comparison of Il-17A in children with IBD depending on the activity of the disease.

The comparison of IL-17A concentration between patients in active phase of IBD and in IBD remission is presented in Fig. 1. We found that serum IL-17A was significantly increased in patients with active phase of ulcerative colitis compared to those in ulcerative colitis remission (p = 0.04). However, there were no significant differences in IL-17A among patients with active Crohn’s disease and in Crohn’s disease remission (p = 0.18).

Moreover, in patients with ulcerative colitis IL-17A demonstrated a positive correlation with stool calprotectin level (p = 0.06; R = 0.31) and PUCAI (p = 0.05; R = 0.31), which almost attained statistical significance.There were no significant correlations between IL-17A and other inflammatory markers i.e. CRP, ESR, white blood cells count. We found negative correlations between IL-17A and haemoglobin level (p = 0.02; R = − 0.36), haematocrit (p = 0.002; R = − 0.47) and medium cell value (MCV) (p = 0.01; R = − 0.36) in ulcerative colitis.

There were no significant correlations between IL-17A and inflammatory markers, PCDAI or red blood cells indices in Crohn’s disease patients.

Although IL-17A concentration was higher in treatment naïve patients (mean ± SD: 25.94 ± 41.18 pg/ml; median: 13.64 pg/ml; range: 0.65–200.54 pg/ml) compared to those on any IBD treatment (mean ± SD: 14.14 ± 13.84 pg/ml; median: 8.86 pg/ml; range: 1.09–58.56 pg/ml), the difference was not statistically significant (p = 0.23).

Serum concentration of IL-17F did not differ significantly between children with IBD (median: 15.11 pg/ml; range: 0.09–189.84 pg/ml; mean ± SD:19.26 ± 23.92 pg/ml) and controls (median: 11.56 pg/ml; range: 0.19–32.49 pg/ml; mean ± SD: 13.52 ± 8.50 pg/ml) (p = 0.33). Table 2 presents comparison of IL-17F serum concentration among children with ulcerative colitis, Crohn’s disease and control group. Concentration of IL-17F did not differ significantly between these subgroups (p = 0.35).

**Table 2:** Serum concentration of IL-17F in children with IBD and controls.

		Ulcerative colitis	Crohn’s disease	Control group	Kruskal–Wallis test
Il-17F (pg/ml)	Mean ± SD	15.45 ± 8.56	25.87 ± 37.55	13.52 ± 8.50	H = 2.09 p = 0.35
	Median (range)	14.16 (0.09–41.75)	15.11 (1.73–189.84)	11.56 (0.19–32.49)	

**Figure 2:**
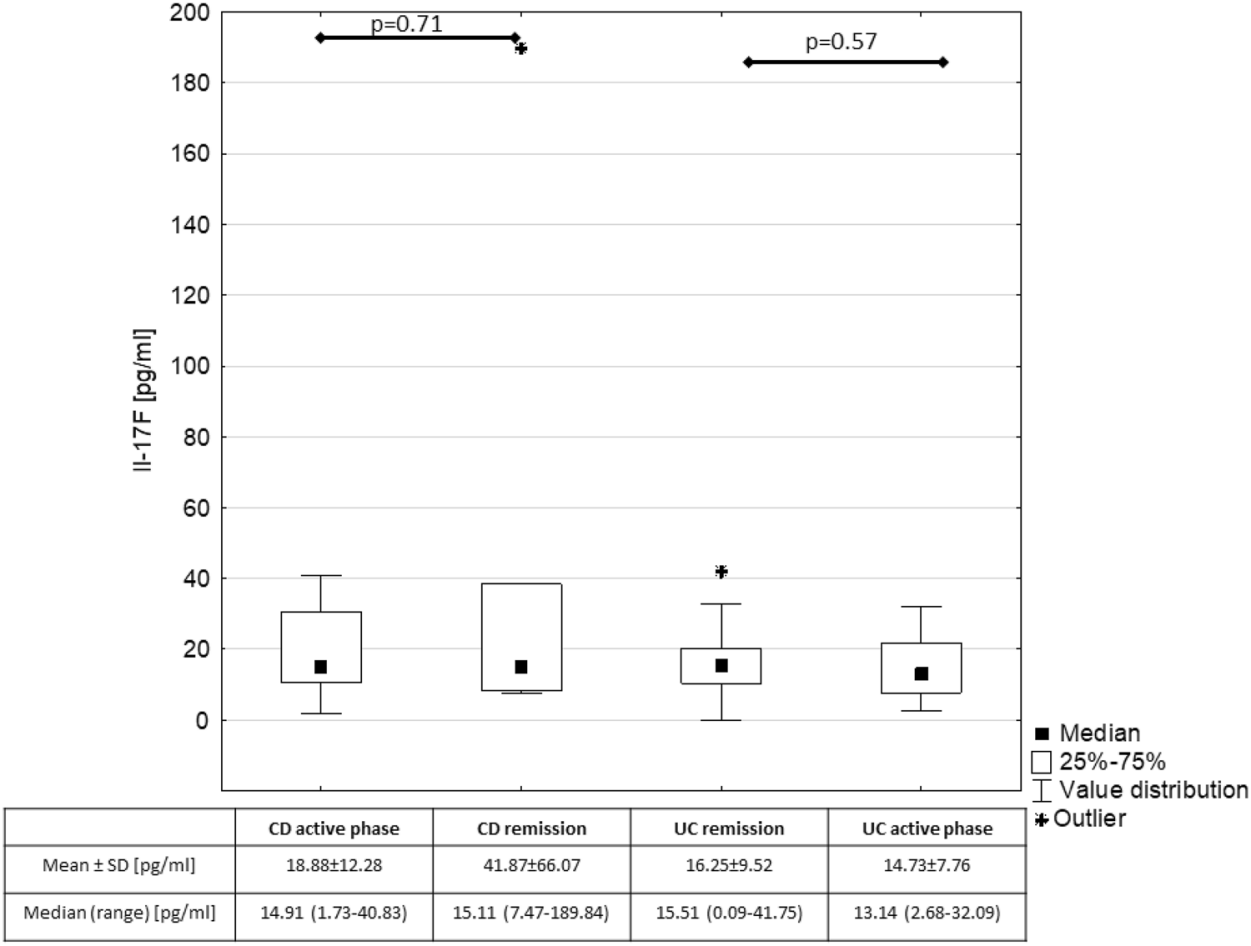
Comparison of Il-17F in children with IBD depending on the activity of the disease.

We found a positive correlation between IL-17A and IL-17F in children with IBD (p = 0.03; R = 0.29). However, there were no significant correlations between IL-17F and IBD activity indices, inflammatory markers i.e. calprotectin, CRP, ESR, white blood cells count. The comparison of serum IL-17F between patients in active phase of IBD and in IBD remission is presented in Fig. 2.

There were no significant differences in IL-17F among treatment naïve patients (mean ± SD: 14.65 ± 10.57 pg/ml; median: 11.93 pg/ml; range: 1.73–40.84 pg/ml) compared to those on IBD therapy (mean ± SD: 21.90 ± 28.75 pg/ml; median: 16.89 pg/ml; range: 0.09–189.84 pg/ml) (p = 0.09).

## Discussion

Considerable progress in the understanding of IBD pathogenesis in recent years entails substantial advances in the diagnosis and therapy of that disease^1,15–18^. However, still there is a need of reliable non-invasive or minimally invasive biomarkers which would facilitate timely recognition of IBD and would predict exacerbation of the disease. It is particularly important in children and adolescent to avoid repeated gastrointestinal tract endoscopy under anaesthesia. It appears that cytokines involved in the inflammatory process underlying IBD may become potential diagnostic tools and moreover represent a target for therapeutic interventions in IBD^6^.

One of cytokines which has been recently explored in terms of IBD pathogenesis is IL-17A. It has been suggested that in the gut antigen-stimulated dendritic cells secrete IL-23 which promotes the differentiation of naive CD4 + T cells into Th-17-cells producing IL-17A^22,23^. IL-17A upregulates intercellular adhesion molecule-1 on intestinal epithelial cells and induces release of IL-6 and IL-8 from epithelial cells and myofibroblasts^23^. It results in an augmented recruitment of neutrophils and production of pro-inflammatory mediators eventually leading to the damage of gut epithelium^22,23^.

Although several studies have been focused on the role of IL-17 family in the pathogenesis of IBD, most of them has been carried out in relatively small populations of patients giving conflicting results^13,14,19–21^. Therefore, to date all the pieces of the puzzle have not been assembled.

In our study we demonstrated that serum concentration of IL-17A was significantly increased in children with IBD compared to the control group. Moreover, in a group of patients with ulcerative colitis relationship between IL-17A and clinical activity index (PUCAI) and calprotectin nearly reached a statistically significant level. On the contrary, there were no significant differences in serum concentration of IL-17F between IBD patients and controls. IL-17F revealed only a single significant correlation with IL-17A.

Our findings indicating higher levels of serum IL-17A in children with IBD than in control group are consistent with some previous studies^20,21^. Kaplan et al. showed significant increase of serum IL-17A in adult patients with IBD compared with the control group^21^. This result ties well with a previous study by Fujino et al.^20^ who presented that serum IL-17A was significantly elevated in a group of adults with IBD compared to controls and to patients with infectious colitis, or ischaemic colitis patients^20^. Present findings along with prior studies support conception of significant role of IL-17A in IBD.

In contrast to these findings Kleiner et al. presented that serum IL-17A was significantly decreased in children with IBD patients compared to healthy controls^14^.

Detailed analysis revealed that in our study group only in patients with ulcerative colitis IL-17A was higher than in controls. Cho et al. did not show any significant differences in serum IL-17A between children Crohn’s disease or ulcerative colitis compared to healthy controls^13^. Moreover, study by Sahin et al. also did not revealed significant differences in serum IL-17A among patients with Crohn’s disease and controls^19^. It may be explained by the fact that patients with Crohn’s disease in these studies had relatively moderate activity of the disease. It might be also related with the effect of IBD therapy leading to inhibition of pro-inflammatory cytokines^19^. Different expression of IL-17A in patients with Crohn’s disease and ulcerative colitis may be associated with the fact that these diseases differ in some aspect of pathogenesis^24^. Kobayashi et al.^25^ found that IL-23 differentially regulates Th1/Th17 balance in ulcerative colitis and Crohn’s disease enhancing production of different cytokines. Thus, ulcerative colitis may be assumed as Th17 disease with increased production of Il-17, while Crohn’s disease as Th1 disease with upregulated synthesis of IFNγ^25^.

In general, conflicting results of studies evaluating IL-17A level may be caused by some discrepancies between studied populations including various disease duration, phenotype, activity, use of diverse medications and concomitant diseases.

We demonstrated that IL-17A may be associated with ulcerative colitis activity which accords with other research^20,21^. Similarly to our findings, serum concentration of IL-17A was significantly increased in active ulcerative colitis patients compared to those in the disease remission in studies conducted by Kaplan et al. and Fujino et al.^20,21^. Moreover, Ohman et al. found that serum IL-17A in newly diagnosed treatment naïve patients with ulcerative colitis correlated with disease severity and predicted the course of the disease over the following three years^26^.

However, in contrast to these studies we did not find significant differences in IL-17A among Crohn’s disease patients depending on the disease activity^20,21^. It may be explained due to various criteria of patients’ qualification into active and non-active IBD group used in these studies.

On the other hand, Sahin et al. revealed no differences in IL-17A between patients with active and inactive Crohn’s disease and significantly decreased level of IL-17A in patients with active Crohn’s disease compared to the control group^19^.

In our study in ulcerative colitis children positive relationship between IL-17A with PUCAI and stool calprotectin showed a tendency toward significance. Kaplan et al. found in all patients with IBD a positive correlation between IL-17A and C-reactive protein, endoscopic activity index, and Crohn’s disease activity index^21^. These findings imply that IL-17A may be considered as a marker for inflammatory activity in IBD patients.

Moreover, we found a negative relationship between IL-17A with haemoglobin, haematocrit, and medium cell value in children with ulcerative colitis. It may be explained by the fact that has IL-17 cytokine family members are suggested to be inhibitors of haematopoiesis^27^. IL-17A downregulates erythropoiesis through inhibition of late stage erythroid progenitors (colony-forming unit-erythroid, CFU-E)^28^.

Interleukin 17F is closely related to IL-17A and its role in the development of inflammatory bowel disease remains not well-established. Seiderer et al. found that intestinal IL-17F mRNA expression was increased in active CD but not in ulcerative colitis^29^. Safari et al. presented no differences in IL-17F mRNA expression in peripheral blood mononuclear cells between patients with Crohn’s disease and ulcerative colitis^30^. Meta-analysis performed by Li et al. revealed correlations between *IL-17A* (G197A) and *IL-17F* (7488T/C) genetic polymorphisms and an increase in UC risk^31^. Recently Tang et al. discovered that suppression of IL-17F, but not of IL-17A, suppressed the development of colitis by inducing T_reg_ cells through modification of the intestinal microbiota^32^. In our study there were no significant differences in serum IL-17F levels between children with IBD and healthy controls. However, there was a positive correlation between IL-17A and IL-17F in children with IBD. This may suggest that although both cytokines share high level of homology at the amino acid level, cellular sources and some functions, IL-17A more strongly than IL-17F is involved in mediating autoimmunity and chronic inflammation, which has been observed previosuly^33^.

The main limitation of our study is relatively homogenic study population with rather moderate clinical activity of the disease. Another limitation may be the fact that patients with ulcerative colitis prevailed in our study group. Moreover, our results should be interpreted with caution since we evaluated the concentration of IL-17A and IL-17F only in patients’ serum. Future research may consider determining the expression of IL-17 in mucosal samples from patients to fully understand correlation between IL-17 family members and IBD activity.

In conclusion we demonstrated that serum IL-17A levels, but not IL-17F, are significantly elevated in children with IBD compared to controls. These results may imply that IL-17A plays critical role in the pathogenesis of paediatric IBD. Moreover, we presented that IL-17A was significantly increased in patients with active ulcerative colitis compared to those in ulcerative colitis remission. Our findings may suggest that IL-17A may be a marker of ulcerative colitis clinical activity in children. Nevertheless, further studies are needed to fully elucidate the contribution of IL-17 family in the pathogenesis of paediatric IBD.

## Methods

### Study population

We recruited consecutive children with IBD hospitalized at the Department of Paediatrics and Gastroenterology, Medical University of Lublin, Poland between June 2017 and October 2019. IBD was diagnosed according to the revised Porto criteria^2^. Disease phenotype was established based on the Paris Classification^34^. Clinical activity in children with Crohn’s disease was determined by Paediatric Crohn’s Disease Activity Index (PCDAI) and in children with ulcerative colitis by Paediatric Ulcerative Colitis Activity Index (PUCAI)^35,36^. Exclusion criteria from the study group were lack of informed consent of parents and/or patient aged ≥ 16 years old, any clinical or laboratory signs of acute infection at the time of enrolment, a history of a surgery within the 4 weeks preceding the recruitment.

In the same study period healthy children with functional abdominal pain were enrolled to the control group. The diagnosis of functional abdominal pain was established on the basis of Rome IV Diagnostic Criteria for Functional Gastrointestinal Disorders^37^. Exclusion criteria were as follows lack of informed consent of parents and/or patient aged ≥ 16 years old, any concomitant chronic disease, any clinical or laboratory signs of acute or chronic inflammation at the time of enrolment, a history of a surgery within the 4 weeks preceding the recruitment.

### Methods

Children underwent routine blood testing including complete blood count, C-reactive protein (CRP), erythrocyte sedimentation rate (ESR) and stool sampling for calprotectin according to standard laboratory practice. Serum concentration interleukin 17A and 17F was measured using commercially available enzyme-linked immunosorbent assay (ELISA) kits (Human IL-17A and Human IL-17F ELISA kits) according to the manufacturer’s recommendations (Diaclone SAS Besancon Cedex, France). According to the instruction IL-17A standard concentration using the standard curve ranged from 0 to 100 pg/ml, while IL17F from 0–500 pg/ml. Following the instructions of the protocol the concentration from the standard curve was multiplied by the appropriate dilution factors.

### Statistical analysis

The statistical analysis was performed using Statistica v. 13 software (StatSoft, Poland). The data are shown as mean and standard deviation or median and range^38,39^. Non-parametric tests for analysis considering skewed distribution of variables (W Shapiro–Wilk test) and inhomogeneity of variance (F-Fisher test) were carried out^38,39^. Differences between two groups were tested with the use of Mann–Whitney *U*-rank test, while differences between more than two means for more than two groups with The H Kruskal–Wallis test^38,39^. Correlations between parameters were expressed by the Spearman’s rank correlation coefficient^38,39^. The results were considered statistically significant at *p* < 0.05^38,39^.

### Compliance with ethical standards

Written informed consent for participation in this study was provided by a parent and also by a patient in case of a child aged ≥ 16 years. The study was approved by the Bioethical Committee of Medical University of Lublin (KE-0254/289/2016). The study was performed in accordance with the 1975 Declaration of Helsinki (6th revision, 2008).

## Data Availability

The datasets generated during and/or analysed during the current study are available from the corresponding author on reasonable request.

## References

[1] Kim DH, Cheon JH (2017). Pathogenesis of inflammatory bowel disease and recent advances in biologic therapies. Immune Netw..

[2] Levine A (2014). ESPGHAN revised porto criteria for the diagnosis of inflammatory bowel disease in children and adolescents. J. Pediatr. Gastroenterol. Nutr..

[3] Geremia A, Biancheri P, Allan P, Corazza GR, Di Sabatino A (2014). Innate and adaptive immunity in inflammatory bowel disease. Autoimmun. Rev..

[4] Zhang YZ, Li YY (2014). Inflammatory bowel disease: Pathogenesis. World J. Gastroenterol..

[5] Cătană CS (2015). Contribution of the IL-17/IL-23 axis to the pathogenesis of inflammatory bowel disease. World J. Gastroenterol..

[6] Katsanos KH, Papadakis KA (2017). Inflammatory bowel disease: Updates on molecular targets for biologics. Gut Liver.

[7] Miossec P (2017). Update on interleukin-17: A role in the pathogenesis of inflammatory arthritis and implication for clinical practice. RMD Open.

[8] McGeachy MJ, Cua DJ, Gaffen SL (2019). The IL-17 family of cytokines in health and disease. Immunity.

[9] Monin L, Gaffen SL (2018). Interleukin 17 family cytokines: Signaling mechanisms, biological activities, and therapeutic implications. Cold Spring Harb. Perspect. Biol..

[10] Rouvier E, Luciani MF, Mattéi MG, Denizot F, Golstein P (1993). CTLA-8, cloned from an activated T cell, bearing AU-rich messenger RNA instability sequences, and homologous to a herpesvirus saimiri gene. J. Immunol..

[11] Moseley TA, Haudenschild DR, Rose L, Reddi AH (2003). Interleukin-17 family and IL-17 receptors. Cytokine Growth Factor Rev..

[12] Kolls JK, Lindén A (2004). Interleukin-17 family members and inflammation. Immunity.

[13] Cho J (2018). Mucosal immunity related to FOXP3 regulatory T cells, Th17 cells and cytokines in pediatric inflammatory bowel disease. J. Korean Med. Sci..

[14] Kleiner G (2015). Pediatric patients with inflammatory bowel disease exhibit increased serum levels of proinflammatory cytokines and chemokines, but decreased circulating levels of macrophage inhibitory protein-1β, interleukin-2 and interleukin-17. Exp. Ther. Med..

[15] Peters LA (2017). A functional genomics predictive network model identifies regulators of inflammatory bowel disease. Nat. Genet..

[16] Nishida A (2018). Gut microbiota in the pathogenesis of inflammatory bowel disease. Clin. J. Gastroenterol..

[17] Guan Q (2019). A comprehensive review and update on the pathogenesis of inflammatory bowel disease. J. Immunol. Res..

[18] Daniluk U (2019). Untargeted metabolomics and inflammatory markers profiling in children with Crohn's disease and ulcerative colitis-A preliminary study. Inflamm. Bowel Dis..

[19] Sahin A (2014). Serum interleukin 17 levels in patients with Crohn's disease: Real life data. Dis. Markers.

[20] Fujino S (2003). Increased expression of interleukin 17 in inflammatory bowel disease. Gut.

[21] Kaplan M (2016). Are sTWEAK and IL-17A levels in inflammatory bowel disease associated with disease activity and etiopathogenesis?. Inflamm. Bowel Dis..

[22] Iwakura Y, Ishigame H (2006). The IL-23/IL-17 axis in inflammation. J. Clin. Invest..

[23] Hohenberger M, Cardwell LA, Oussedik E, Feldman SR (2018). Interleukin-17 inhibition: Role in psoriasis and inflammatory bowel disease. J. Dermatol. Treat..

[24] Miossec P (2009). IL-17 and Th17 cells in human inflammatory diseases. Microbes Infect..

[25] Kobayashi T (2008). IL23 differentially regulates the Th1/Th17 balance in ulcerative colitis and Crohn's disease. Gut.

[26] Ohman L (2013). Serum IL-17A in newly diagnosed treatment-naive patients with ulcerative colitis reflects clinical disease severity and predicts the course of disease. Inflamm. Bowel Dis..

[27] Broxmeyer HE (2006). The IL-17 cytokine family members are inhibitors of human hematopoietic progenitor proliferation. Blood.

[28] Mojsilović S, Jauković A, Santibañez JF, Bugarski D (2015). Interleukin-17 and its implication in the regulation of differentiation and function of hematopoietic and mesenchymal stem cells. Mediators Inflamm..

[29] Seiderer J (2008). Role of the novel Th17 cytokine IL-17F in inflammatory bowel disease (IBD): upregulated colonic IL-17F expression in active Crohn's disease and analysis of the IL17F p.His161Arg polymorphism in IBD. Inflamm. Bowel Dis..

[30] Safari MT (2017). Evaluation of IL-17B and IL-17F mRNA expression in peripheral blood mononuclear cells and association with clinical outcome of IBD patients. Gastroenterol. Hepatol. Bed Bench.

[31] Li J, Tian H, Jiang HJ, Han B (2014). Interleukin-17 SNPs and serum levels increase ulcerative colitis risk: A meta-analysis. World J. Gastroenterol..

[32] Tang C (2018). Suppression of IL-17F, but not of IL-17A, provides protection against colitis by inducing T. Nat. Immunol..

[33] Ishigame H (2009). Differential roles of interleukin-17A and -17F in host defense against mucoepithelial bacterial infection and allergic responses. Immunity.

[34] Levine A (2011). Pediatric modification of the Montreal classification for inflammatory bowel disease: The Paris classification. Inflamm. Bowel Dis..

[35] Hyams JS (1991). Development and validation of a pediatric Crohn's disease activity index. J. Pediatr. Gastroenterol. Nutr..

[36] Turner D (2007). Development, validation, and evaluation of a pediatric ulcerative colitis activity index: A prospective multicenter study. Gastroenterology.

[37] Hyams JS (2016). Functional disorders: Children and adolescents. Gastroenterology.

[38] Krawiec P, Pac-Kożuchowska E (2019). Soluble transferrin receptor and soluble transferrin receptor/log ferritin index in diagnosis of iron deficiency anemia in pediatric inflammatory bowel disease. Dig. Liver Dis..

[39] Krawiec P, Mroczkowska-Juchkiewicz A, Pac-Kożuchowska E (2017). Serum hepcidin in children with inflammatory bowel disease. Inflamm. Bowel Dis..

